# Adherence to and effectiveness of an individually tailored home-based exercise program for frail older adults, driven by mobility monitoring: design of a prospective cohort study

**DOI:** 10.1186/1471-2458-14-570

**Published:** 2014-06-07

**Authors:** Hilde AE Geraedts, Wiebren Zijlstra, Wei Zhang, Sjoerd Bulstra, Martin Stevens

**Affiliations:** 1 Center for Human Movement Sciences, University of Groningen, University Medical Center Groningen, Groningen, Netherlands; 2 Institute of Movement and Sport Gerontology, German Sport University Cologne, Cologne, Germany; 3 Philips Research Europe, Eindhoven, Netherlands; 4 Department of Orthopaedics, University of Groningen, University Medical Center Groningen, Groningen, Netherlands

**Keywords:** Home-based physical activity program, Remote coaching, Daily activity, Objective measurement, Frail older adults

## Abstract

**Background:**

With the number of older adults in society rising, frailty becomes an increasingly prevalent health condition. Regular physical activity can prevent functional decline and reduce frailty symptoms. In particular, home-based exercise programs can be beneficial in reducing frailty of older adults and fall risk, and in improving associated physiological parameters. However, adherence to home-based exercise programs is generally low among older adults. Current developments in technology can assist in enlarging adherence to home-based exercise programs. This paper presents the rationale and design of a study evaluating the adherence to and effectiveness of an individually tailored, home-based physical activity program for frail older adults driven by mobility monitoring through a necklace-worn physical activity sensor and remote feedback using a tablet PC.

**Methods/design:**

Fifty transitionally frail community-dwelling older adults will join a 6-month home-based physical activity program in which exercises are provided in the form of exercise videos on a tablet PC and daily activity is monitored by means of a necklace-worn motion sensor. Participants exercise 5 times a week. Exercises are built up in levels and are individually tailored in consultation with a coach through weekly telephone contact.

**Discussion:**

The physical activity program driven by mobility monitoring through a necklace-worn sensor and remote feedback using a tablet PC is an innovative method for physical activity stimulation in frail older adults. We hypothesize that, if participants are sufficiently adherent, the program will result in higher daily physical activity and higher strength and balance assessed by physical tests compared to baseline. If adherence to and effectiveness of the program is considered sufficient, the next step would be to evaluate the effectiveness with a randomised controlled trial. The knowledge gained in this study can be used to develop and fine-tune the application of innovative technology in home-based exercise programs.

**Trial registration:**

Nederlands Trial Register (NTR); trial number 4265. The study was prospectively registered (registration date 14/11/2013).

## Background

As the number of older adults in our society rises [1], health professionals, including physiotherapists, are increasingly confronted with frail older adults. Frailty is a multidimensional feature that has been defined and described in many different ways. A widely used definition is that of Fried [2]: “Frailty is the state of vulnerability to stressors that is independent of any specific disease or disability but that is common in older people and predisposes them to various adverse health outcomes”. Frailty is an important predisposition for falls and associated adverse health conditions [3]. Previous research indicates that regular physical activity has many beneficial effects on daily functioning, balance and strength, as well as other health-related factors in older adults [4,5]. Exercise programs can potentially be helpful in reducing frailty of older adults and fall risk, and in improving associated physiological parameters, thus facilitating a longer independent life [6]. Specifically, home-based physical activity programs can be considered promising in the promotion of a physically active lifestyle among frail older adults [7]. Daily physical activity and adherence to home-based exercise programs is generally low among this group though [8]. This compromises the effectiveness of home-based exercise programs.

Current developments in technology can assist physiotherapists in enlarging adherence to home-based exercise programs. The use of objective activity monitoring with wearable sensors can potentially be helpful in strategies aimed at increasing daily activity and adherence to home-based physical activity programs [9]. Recent studies have shown the possibility to monitor mobility-related activities based on a thorax-worn motion sensor [10] or motion sensors on the lower trunk [11]. Such sensor-based approaches can be used to measure physical activity by detecting and monitoring different postures (i.e. lying, sitting, standing) and activities such as rising from a chair and walking. Motion sensing-based activity monitoring combined with information and communication technology (ICT) create the possibility to remotely monitor and influence daily physical activity behaviour in real life, instead of solely under laboratory circumstances or in a physiotherapy practice. Hence technology can assist in enlarging adherence by providing home-based exercise programs through gaining participant information and enabling remote contact with participants. The use of computers and tablets is steadily rising among older adults in the Netherlands [12]. How such new technology can be used to optimally support exercise-based interventions tailored for older adults is not yet clear though.

We recently developed an innovative home-based physical activity intervention that is based on the use of a tablet PC in order to present a home-based exercise program and a necklace-worn motion sensor to continuously monitor mobility-related activities. The data monitoring is used for remote coaching of intervention participants. The current paper presents the design of a study that aims to evaluate the adherence to and effectiveness of our approach on independently living, transitionally frail older adults.

The primary research question is: What is the adherence to a home-based exercise program for use by older adults including exercise instructions from a tablet PC and monitoring by means of a necklace-worn motion sensor, as determined by adherence to the exercise program and wearing of the sensor?

Secondary research questions are: Does participation in the home-based exercise program (including use of the sensor and tablet) increase the daily amount of physical activity, measured both objectively and self-reportedly? Does participation in a home-based exercise program (including use of the sensor and tablet) improve functional performance?

## Methods

### Study design

**Figure 1:**
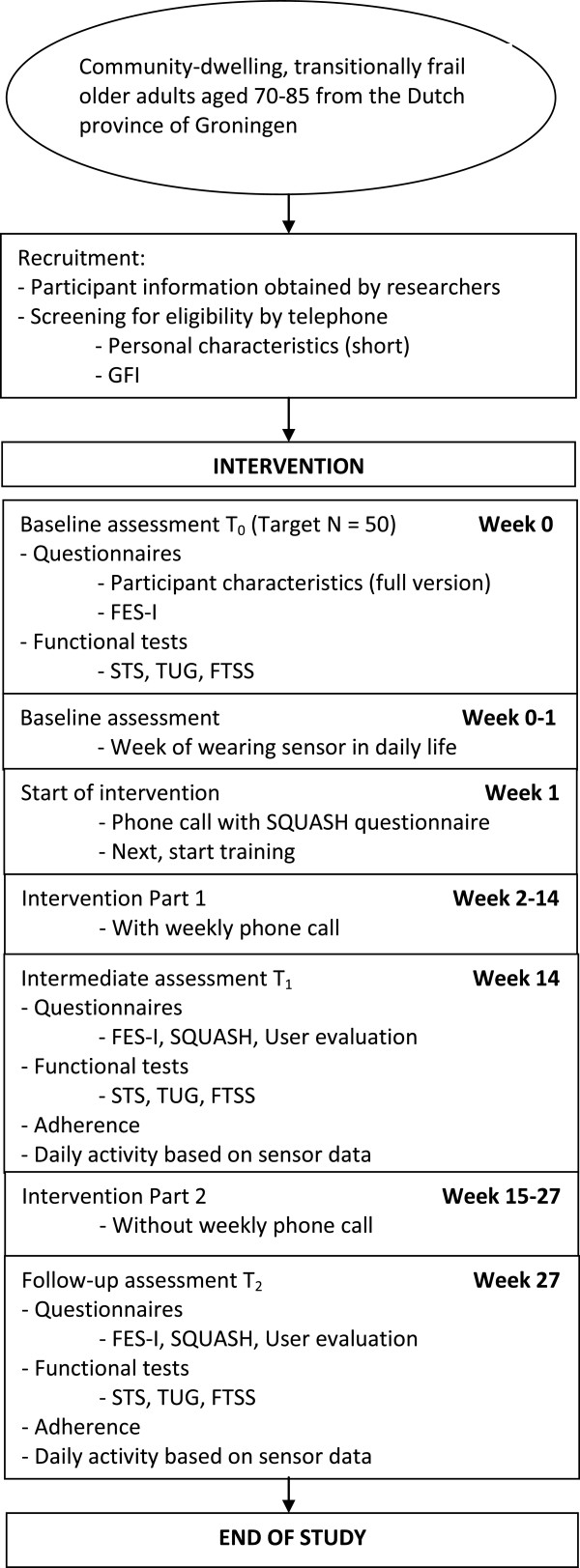
Flow chart of the study design.

This will be a prospective cohort study. Participants will join a 6-month home-based exercise program. Training and measurements will take place at participants’ homes. Study design and procedures are approved by the Medical Ethics Committee of the University Medical Center Groningen (UMCG). See Figure 1 subsection for a flowchart of the study design.

### Participants

Fifty subjects will be included. Inclusion criteria will be:

– Age 70–85 years.

– Transitional frailty as measured by the Groningen Frailty Indicator (GFI). The GFI is a screening instrument for level of frailty. Scores range from zero (not frail) to 15 (very frail). Older adults will be considered transitionally frail if they have a GFI score of 4 or 5. This GFI score indicates persons with only a minor elevated chance of loss of functionality and heightened disability [13].

– Community dwelling or assisted living conditions.

– Ability to walk 10 m without support or using a cane or walker.

– Availability of a telephone.

– Ability to understand instructions regarding the use of the technology and execution of the exercise program.

Persons will be excluded based on conditions that hamper safe execution of the exercise program:

– Total hip or knee replacement in the past 6 months.

– Visual problems to a degree that makes it impossible for the subject to accurately read the questionnaires or walk around safely in his own home.

– Stroke within the last 6 months.

– Parkinson’s disease stage 4 or 5.

– Other neurological conditions that can impair daily functioning (e.g. dementia).

### Recruitment

Recruitment will be done by several means:

1) At information gatherings and when spreading information folders in neighbourhoods where many older people live.

2) Through healthcare organisations for older adults.

3) Recruitment in the participant pool of SamenOud. SamenOud is an UMCG-initiated project introducing a new healthcare model that combines all aspects of older-adult health care: living, well-being and care. SamenOud is running in the north-eastern part of the Dutch province of Groningen [14].

### Intervention

All older adults participate in a home-based exercise program that consists of 3 months of home-based exercising with weekly telephone support from a coach and 3 months follow-up of exercising without coach contact. Participants exercise 5 days a week, starting with exercise bouts of 10 minutes which can progress up to 45 minutes. The program progresses in 18 levels. The first step-in level of the program consists of light and easy exercises, in order to accommodate for sedentary participants. Progression of difficulty and duration of exercises across levels elapses in small steps and in consultation with the coach. Participants are not obliged to finish all levels during the program.

The exercise program is based on the Otago Kitchen Table home-based exercise program, an individually tailored fall prevention program for muscle strengthening and balance-retraining exercises of increasing intensity used worldwide [15,16]. The exercises are functional and closely related to daily activities (e.g. standing up from a chair).

From a behavioural point of view this program is based on insights from Social Cognitive theory [17]. The program aims to enhance self-efficacy. Self-efficacy is defined as “the belief in one’s capabilities to organize and execute the courses of action required to manage prospective situations.” [18]. It is hypothesised that by enhancing self-efficacy adherence to the program will improve. In order to enhance self-efficacy, motivational feedback is provided to the participant. The layout of the program as well as the provided feedback is based on the four main sources of efficacy information described by Bandura in the Social Learning theory. These sources include performance accomplishments, vicarious experience, social persuasion, and physiological and emotional states [18]. The sources are used in four different strategies:

1) Exercises are presented by a role model in the instructional videos.

2) An encouraging message is provided after completing an exercise bout.

3) Automatic feedback on time spent “active” (standing and walking) is provided on a daily basis.

4) Weekly phone calls with the coach will be applied, addressing progression and motivational issues during the first three months.

Two devices are utilised during the program: a sensor to collect daily activity data and a tablet PC to provide exercise instructions and feedback.

#### Mobility monitoring

Daily mobility will be monitored by a necklace-worn motion sensor. The sensor is a miniature hybrid sensor that contains a 3D-MEMS accelerometer together with a barometric pressure sensor. Data is sampled at 50 Hz with a 4 mg resolution. A Micro-SD card will be used for data storage and exchange. The sensor weighs about 30 gr and measures about 55×25×10 mm. Measured sensor data is used to identify different activities, like standing, sitting, lying and walking [10,19,20]. Daily activity level will be calculated based on these categories. Participants will wear the sensor during the daytime and connect it to the tablet PC at night for data upload and battery reloading.

#### Tablet PC

A tablet PC is provided as a user device to give exercise instructions and distant feedback. Functionality of the tablet PC is adjusted to independent older adult use, keeping menus and necessary interaction as simple as possible. Completion of the exercise bouts is notified through the tablet PC. Participants are able to choose their level of exercise, after which they are provided with a video showing the entire exercise bout being performed by an older adult, which they are asked to imitate. By means of this procedure they are guided through the entire exercise bout. The videos include a spoken explanation of the exercises while the exercises are being shown. After completing the video, a motivational message based on the rate of completion of the video appears on the screen. The tablet collects data on number of shown videos and the level of the videos shown. Feedback on daily activity level as registered by the sensor is provided by means of a graph that compares daily activity performance to one’s earlier performances as well as to a norm population.

### Outcome measures

Demographic characteristics (e.g. age, gender, family status and comorbidity) and information on experience with computers, tablets and smartphones will be collected at baseline. Also, written informed consent will be completed at baseline. In addition to the monitoring data, standardised measurements will be taken at baseline (T_0_), at 3 months (T_1_) and at 6 months (T_2_).

#### Primary outcome measure

*Adherence*: Adherence to the intervention will firstly be assessed based on adherence to the exercise program and wearing of the sensor. Adherence to the exercise program will be calculated based on completion of exercise bouts. Adherence to wearing the sensor will be calculated based on the number of days the sensor is worn and the successful collection of data. Adherence will be considered sufficient when adherence to the exercise program and to the wearing of the sensor exceeds 70%.

Additional information on factors that may influence adherence will be collected by means of a questionnaire. The user evaluation questionnaire is an adapted version of the SensAction-AAL subject evaluation form [21]. The questionnaire contains questions about the perceived burden of the intervention, wearing of the sensor, and acceptability of the technology. Information will be collected at T_1_ and T_2_.

#### Secondary outcome measures

*Objective daily physical activity based on sensor data*: Baseline (T_0_) information on time spent “active” (standing and walking) will be collected while wearing the sensor for a week before starting the exercise program. A week of wearing will also be used as intermediate and follow-up information on daily activity at T_1_ and T_2_.

*Self-reported daily physical activity*: In addition to objective daily physical activity, self-reported daily physical activity will be measured by questionnaire at T_0_, T_1_ and T_2_. The Short Questionnaire to Assess Health-enhancing physical activity (SQUASH) gives an insight into habitual physical activity [22]. The SQUASH consists of four domains: A) commuting activities, B) leisure-time activities, C) household activities, and D) activities at work and school. The questions within the four domains are prestructured into frequency, duration and intensity of an activity. Reproducibility and relative validity of the SQUASH for assessing physical activity have been evaluated [22,23].

*Functional performance*: Functional performance is assessed by means of several clinical tests for physical functioning at T_0_, T_1_ and T_2_. First, subjects are requested to stand up from a chair at a self-selected pace (Sit-To-Stand; STS). STS is commonly used as a test to assess lower-limb strength and balance [24,25]. Slower test scores are associated with higher fall risk in older adults [26]. Subjects are instructed to sit still for several seconds, stand up from the chair at their preferred pace and then stand still for several seconds. The STS movement has good inter-rater and test-retest reliability in healthy older adults [27]. The test is repeated three times if possible.

Second, the Timed Up-and-Go test (TUG) will be used to measure balance and functional mobility. The TUG is a widely used clinical test and is known to be associated with fall risk in older adults [28,29]. The test protocol includes standing up from a chair, walking 3 m at a preferred speed, turning 180°, walking back and sitting down again. Intra-rater reliability ICC as well as inter-rater reliability ICC of the TUG are excellent [30]. The test is deemed to be a valid measure of dynamic balance and functional control in older adults [31]. This test will be repeated two times if possible.

Third, the Five-Times-Sit-to-Stand test (FTSS) will be used to indicate postural control and lower extremity strength. The FTSS has been associated with postural control, lower extremity strength and fall risk in older adults [26]. Slower test scores have been related to more functional impairments in daily living among older adults [32]. Subjects are instructed to stand up from a chair consecutively five times as fast as possible, preferably without using their hands for support. Test-retest reliability of the FTSS in community-dwelling older adults is adequate [33,34]. The FTSS is deemed to be a valid measure of dynamic balance and functional ability in community-dwelling older adults [31]. Inter-rater reliability is good to excellent [34,35]. The test will be repeated twice if possible.

In addition, subjects follow a weekly functional performance self-assessment protocol independently while wearing the sensor. The protocol consists of one STS, one TUG and one FTSS test. Subjects are asked to shake their sensor five times before and after performing their self-assessment, to mark the self-assessment in their daily life data.

### Data acquisition

Demographics collected by written questionnaire and information on adherence and personal circumstances collected during telephone contacts are manually entered into a database. Information on daily physical activity during the week of wearing and daily life data are collected by the worn sensor. These data are collected during the week before the home visits at T_0_, T_1_ and T_2._ Functional tests are performed during the home visits and by self-assessment; data from the functional tests are collected by using the sensor as well as by registration of duration using a stopwatch. For self-assessment, subjects note the date and time at which they perform the weekly self-assessment and shake the sensor five times before and after to mark the self-assessment in the daily life data. All sensor data are transmitted to a tablet by Bluetooth every night and then stored in a web-based interface. Stopwatch times are entered manually into the database.

### Data analysis

Descriptive statistics will be used to describe the baseline and demographic characteristics as well as data on adherence. Changes in daily physical activity and performance on the functional performance tests at T_0_, T_1_ and T_2_ will be analysed by means of Repeated measures ANOVA. Subgroup analyses will be conducted taking into account the potential influence of age, gender, marital status, GFI and BMI. All data will be analysed with Matlab (version 2013a) and SPSS (version 20.0). A p-value lower than 0.05 will be considered statistically significant.

#### Sample size calculation

Sample size calculation is based on the secondary outcome measure of objective physical activity behaviour as measured with the sensor. Based on earlier studies on the effectiveness of physical activity programs for older adults, it should be possible to reach a 15% increase in objective daily activity within six months [36]. To detect this difference with 80% power (significance level 0.05), a group of 40 participants completing the entire program is required. Assuming a dropout rate of 20%, a total of 50 participants should be enough to demonstrate an increase in daily activity.

## Discussion

The objective of this study is to investigate adherence to and determine the effectiveness of an intervention using a necklace-worn sensor and a tablet PC for driving an individualised home-based physical activity program for transitionally frail older adults.

The optimal design for home-based physical activity programs for older adults using advanced technology is not yet clear. Adherence to such programs is an important issue [37]. Although a general cut-off point for sufficient adherence has not yet been defined, in the literature a cut-off value of 70% adherence is often used. Adherence to home-based physical activity programs for older adults can be influenced by several factors. First, the physical activity program should be physically attainable and encouraging for the participant. In contrast to many group-based physical activity programs found in literature, home-based physical activity programs provide an opportunity to tailor the exercise to the participant. Individual tailoring is a means to ensure practical attainability [5]. Such customisation can be quite extensive, giving each participant his or her own specific training conditions. It can also be more structured, by providing levels of exercising and letting subjects progress through these levels at their own pace. In this study, structured individual tailoring is provided in order to keep the exercise program well-defined.

Adherence is also influenced by the means through which a program is provided to participants. Technology should be encouraging to the target group [38]. Applications should be easy to use independently by older adults, who are generally limited in their use of modern technology [12]. A major advantage of tablet PCs is the use of touchscreens. Touchscreens are instinctive in their use and provide opportunities for lay users, like older adults. In addition, the body-worn sensors are small, unobtrusive tools that can be used to easily gain objective insight into the amount of physical activity of older adults [9]. Accurate measurement of physical activity is essential for proper feedback to participants and to get an impression of the effectiveness of the intervention.

In conclusion, the strength of the current study is the personal tailoring of the exercise program as well as the individual feedback based on actual objective performance of the participant. The ultimate goal is to make the innovative technology available in the community for older adults and health professionals like physiotherapists.

## Competing interests

The authors declare no competing interests.

## Authors’ contributions

HG, WZ, WZh and MS were responsible for study conceptualization, design and development. HG, WZ and MS drafted the manuscript. All authors read and approved the final manuscript.

## Pre-publication history

The pre-publication history for this paper can be accessed here:

http://www.biomedcentral.com/1471-2458/14/570/prepub
